# Supplementary figures and images for: Human mobility data and machine learning reveal geographic differences in alcohol sales and alcohol outlet visits across U.S. states during COVID-19
Source: PLoS One. 2021 Dec 17;16(12):e0255757. doi: 10.1371/journal.pone.0255757 (PMC8683037; doi:10.1371/journal.pone.0255757)

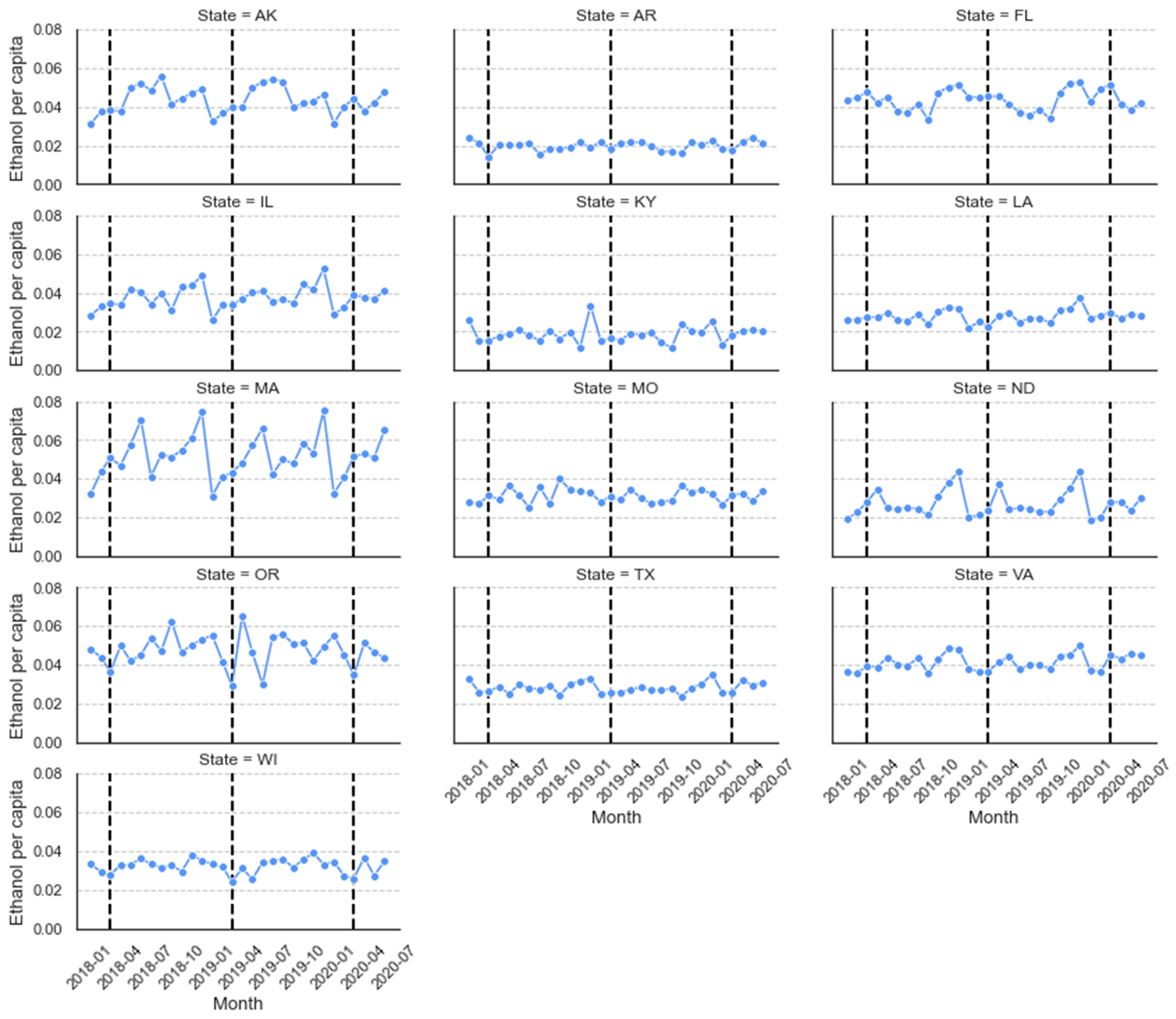

Supplement: S1 Fig — The vertical dashed lines indicate the month of March in 2018, 2019, and 2020. (TIF) [file pone.0255757.s001.tif]

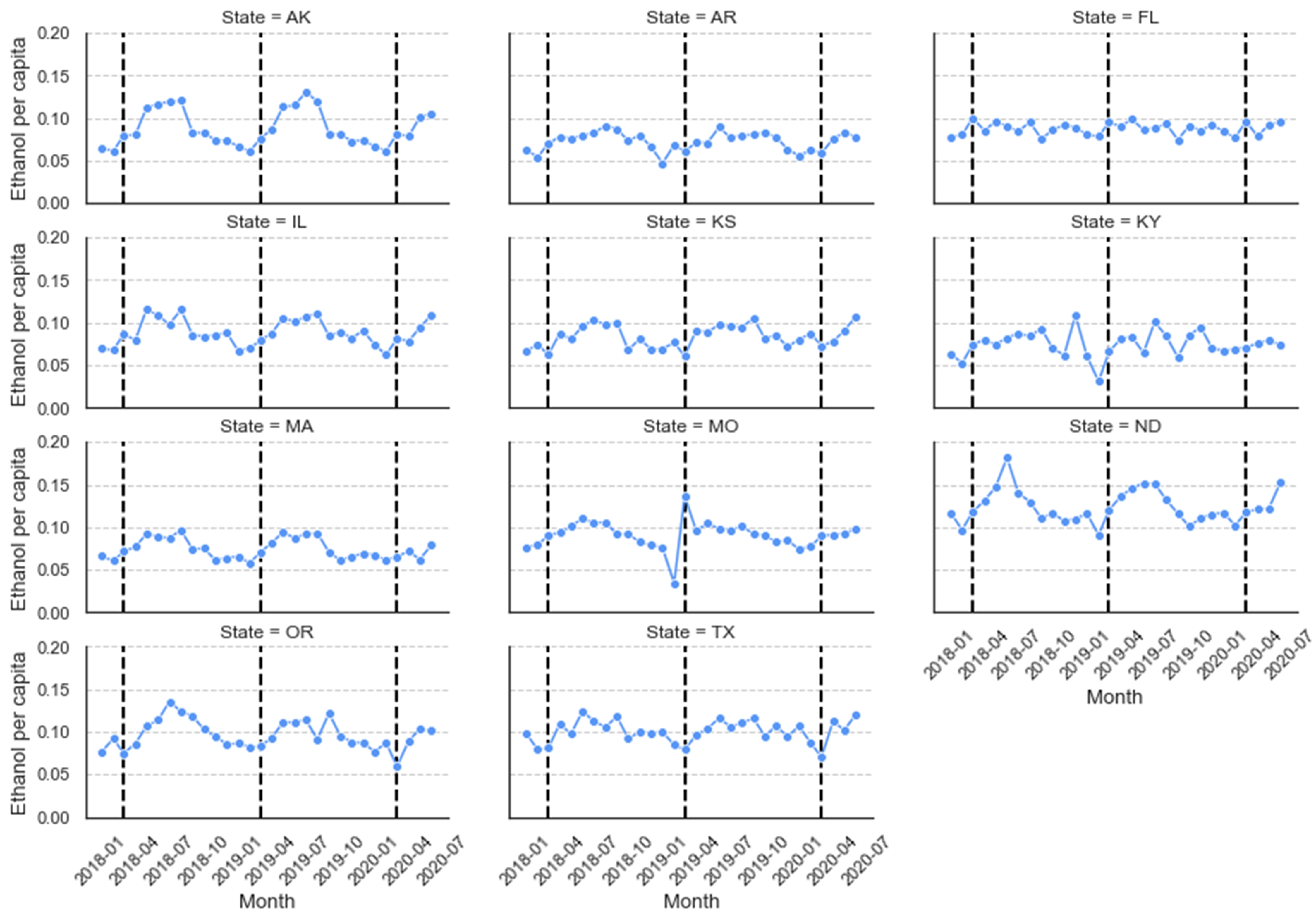

Supplement: S2 Fig — The vertical dashed lines indicate the month of March in 2018, 2019, and 2020. (TIF) [file pone.0255757.s002.tif]

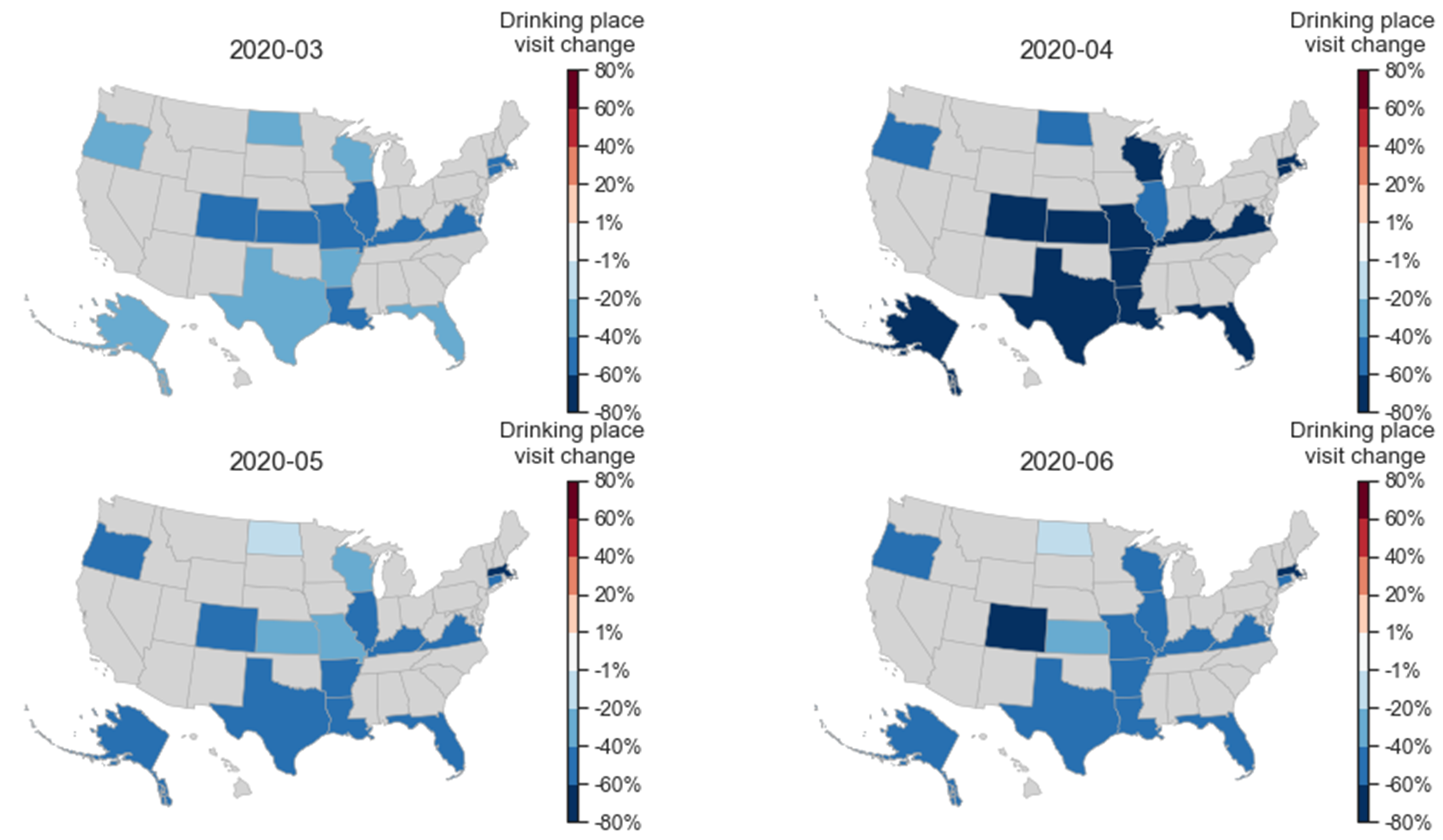

Supplement: S3 Fig — For each month, the percentage change is relative to the average value for the same month in 2018 and 2019. (TIF) [file pone.0255757.s003.tif]

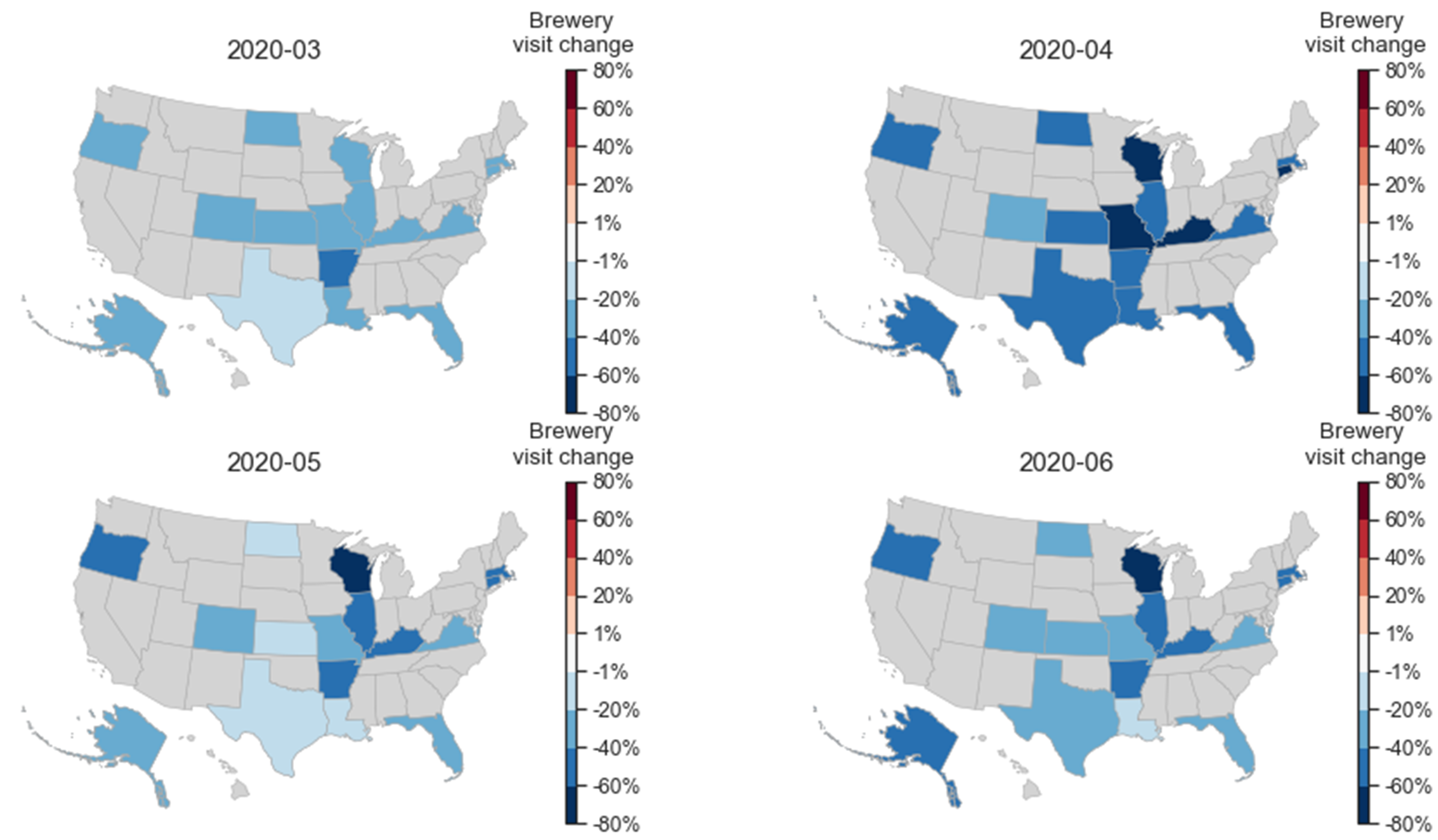

Supplement: S4 Fig — For each month, the percentage change is relative to the average value for the same month in 2018 and 2019. (TIF) [file pone.0255757.s004.tif]

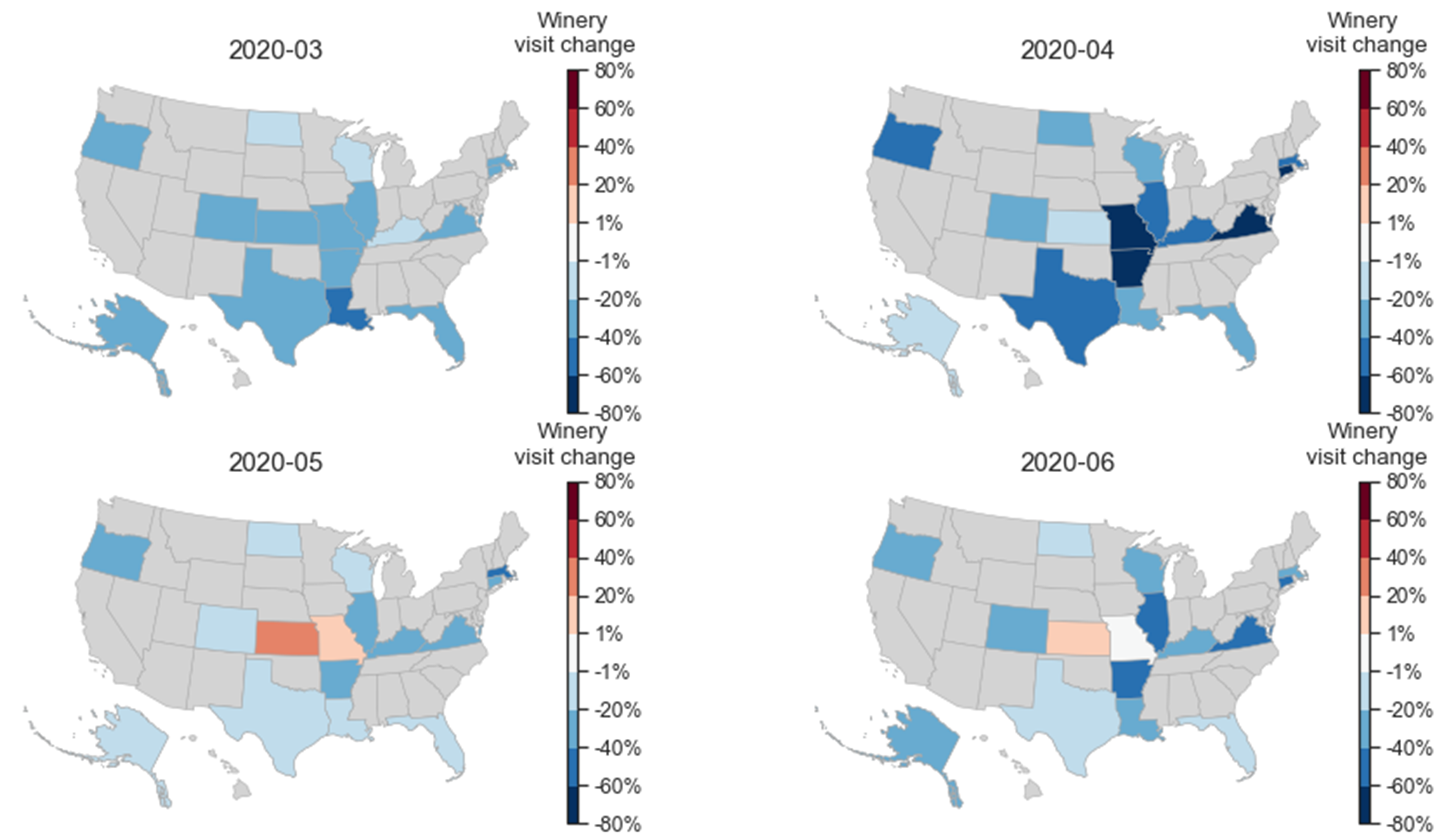

Supplement: S5 Fig — For each month, the percentage change is relative to the average value for the same month in 2018 and 2019. (TIF) [file pone.0255757.s005.tif]

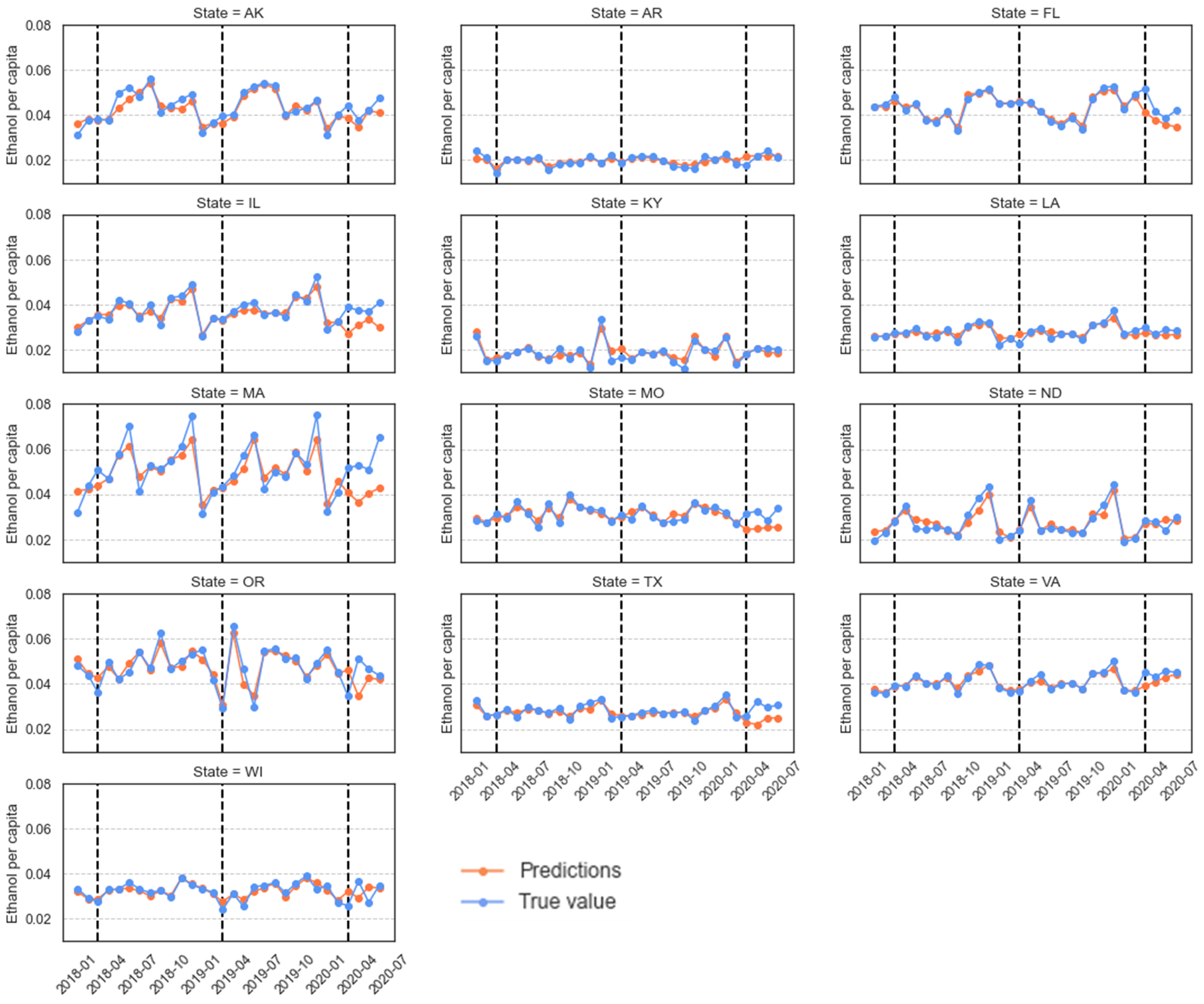

Supplement: S6 Fig — The vertical dashed lines indicate the month of March in 2018, 2019, and 2020. (TIF) [file pone.0255757.s006.tif]

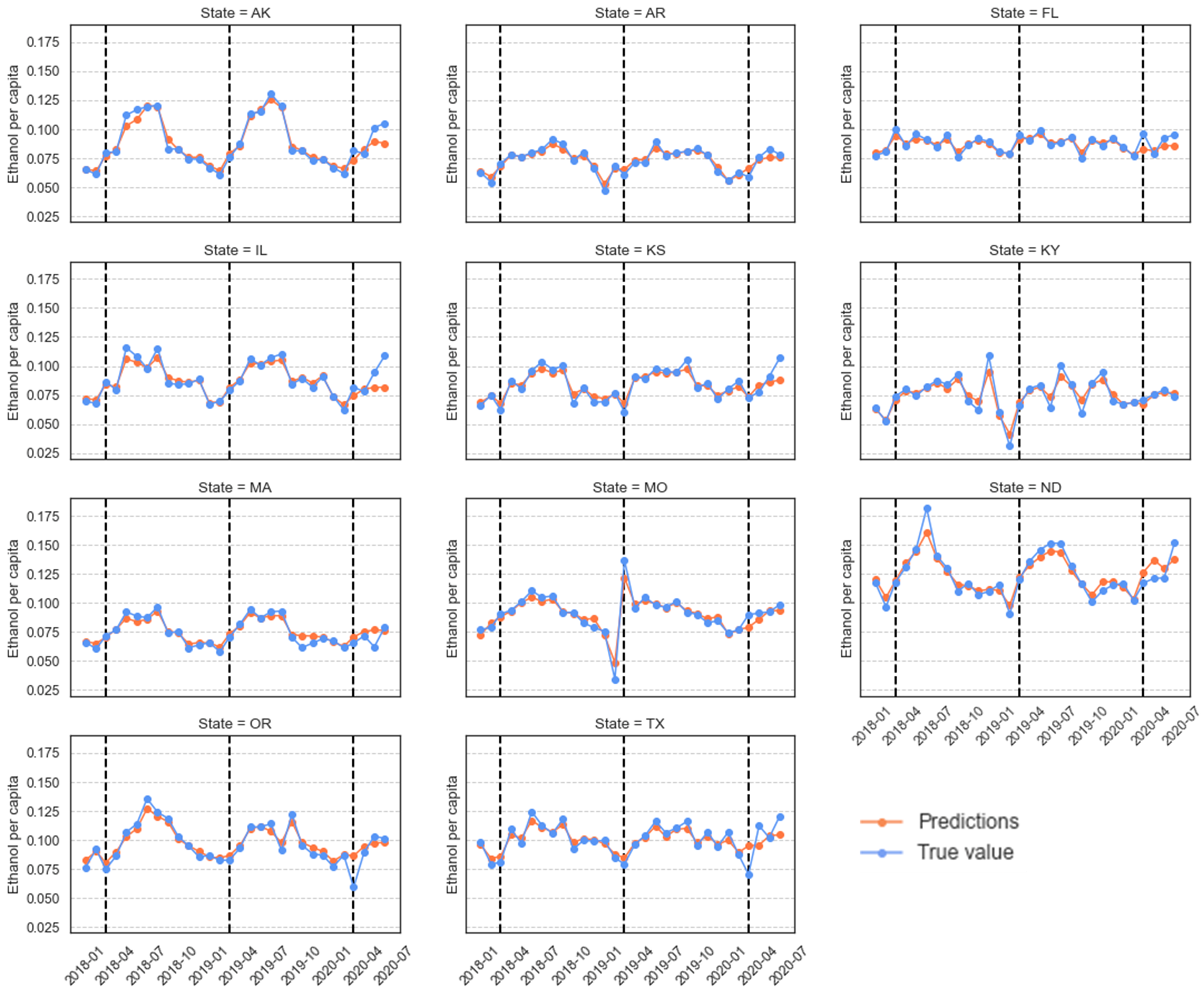

Supplement: S7 Fig — The vertical dashed lines indicate the month of March in 2018, 2019, and 2020. (TIF) [file pone.0255757.s007.tif]
