# Supplementary material for: Human mobility data and machine learning reveal geographic differences in alcohol sales and alcohol outlet visits across U.S. states during COVID-19
Source: PLoS One. 2021 Dec 17;16(12):e0255757. doi: 10.1371/journal.pone.0255757 (PMC8683037; doi:10.1371/journal.pone.0255757)
Supplement: Attachment — Submitted filename: PLOS One.docx [file pone.0255757.s008.docx]

Review for **Human mobility data and machine learning reveal geographic differences in U.S. alcohol sales and alcohol outlet visits during COVID-19**

**(ID:** PONE-D-21-09766**)**

The authors examined alcohol sales and alcohol outlet visits in 16 U.S. states using surveillance reports from the National Institute on Alcohol Abuse and POI visits from various alcohol outlets released by SafeGragh, a commercial company. They further examined geographic differences in changes to alcohol sales and outlet visits during the COVID-19 stay-at-home period. Their results suggest increases in sales of spirits and wine since March 2020, while decreases in the sales of beer. This manuscript is written in great English and is easy to follow. However, the reviewer observed several major limitations that impact the scientific value of this research.

1. The authors only investigated 16 U.S. states in U.S. (out of 50) due to data limitations. Several heavily populated states, such as NY and CA, are not even included. Such a limitation greatly reduces the scientific value of this research, given the small and presumably biased spatial coverage of their data. The author mentioned “geographic difference” many times (also appeared in their title). However, besides presenting the spatial distribution in maps, no spatial algorithm is implemented, nor connection to geographic laws is made. The geographic knowledge is presented by the effort of “mapping”, and “mapping” only.
2. The authors selected four types of POI as alcohol outlets in their study, i.e., Liquor Store, Drinking Place, Brewery, and Winery. However, as the authors mentioned, people can still purchase alcohol from other types of POIs, such as grocery stores, which the reviewer believes is actually the major source for alcohol purchase. Due to the pandemic, it is reasonable to assume that people might reduce the diversity of their POI visits (such as reducing visits to Drinking Places, Brewery, etc.) and only keep essential ones. Grocery store visiting is essential, and it is likely people will direct their alcohol purchases in grocery stores. Unfortunately, such a major (influential) alcohol outlet is not covered.
3. The authors tested three algorithms multiple regression, Random Forest, and DNN. For Multiple Regression, no collinearity test is presented to reveal if these input parameters satisfy the underlying independent assumption. For the Random Forest model, no hyperparameter tuning is implemented to search for the best parameter configuration (the author just used 100 trees as default and did not explore other optimization options). The authors did not provide an evaluation of variable importance nor a sensitivity test to reveal how different inputs would influence their prediction.
