# Supplementary material for: Human mobility data and machine learning reveal geographic differences in alcohol sales and alcohol outlet visits across U.S. states during COVID-19
Source: PLoS One. 2021 Dec 17;16(12):e0255757. doi: 10.1371/journal.pone.0255757 (PMC8683037; doi:10.1371/journal.pone.0255757)
Supplement: Attachment — Submitted filename: Response to Reviewers.docx [file pone.0255757.s009.docx]

**Responses to review comments:**

**Reviewer #2:**

The authors enhance the discussion on their data issues. However, the reviewer believes that their dataset that only covers 16 U.S. states out of 50 is a huge mismatch to their title, which is "revealing geographic differences in U.S. alcohol sales and alcohol outlet visits". How can U.S. 16 states (without heavily populated states like NY and CA) represent the entire U.S? If this limitation can not be addressed, at least, the title of this study needs to be modified to correctly reflect the scope of this study. "in (16) selected U.S. States" could be an option. The reviewer is satisfied with other responses.

Our response: We thank the reviewer’s further comment. We carefully considered the suggestion of the reviewer on revising the title to “in (16) selected U.S. States”, but felt that the word “selected” might imply that we deliberately chose a subset of the available data (while we studied all the available data). We eventually revised the title to “Human mobility data and machine learning reveal geographic differences in alcohol sales and alcohol outlet visits across U.S. states during COVID-19”. In addition, our abstract also explicitly states that we study a *subset* of states.

**Reviewer #3:**

Thank you for addressing appropriately to most of the comments given by reviewers. Before I can recommend this paper for publication, there is still one thing I would like to point out regarding the result of this study.

Our response: Many thanks to the reviewer for reading our paper and giving us constructive comments. Please see our item-by-item responses below.

Authors attempted to apply a number of cutting-edge methodology including human mobility data and machine learning in this study, so as to study the association between alcohol sales data and visiting behavior of people to different POIs including alcohol outlets. The result section of this important area remains unclear. In a nutshell, do authors argue that “if more people visit those POIs, it resulted in the increasing of alcohol sale”? (if so, it sounds quite commonsensical) If not, what the authors were trying to say after investigating their associations?

Our response: We thank the reviewer’s comment. Indeed, in a normal situation, more visits to alcohol outlets will likely lead to higher alcohol sales. However, under the special context of COVID-19, higher alcohol sales are not necessarily linked to more alcohol outlet visits, since people may purchase alcohol online in order to reduce their exposure to the virus, or people may purchase an increased amount of alcohol in a single visit. Our study, particularly RQ3, examines how the relation between alcohol sales and alcohol outlet visits might have changed before and during the early months of COVID-19.

In this revision, we clarified this point in Lines 570-584: “While alcohol sales and alcohol outlet visits are naturally related, our study focused on how their relation might have changed under the special context of the COVID-19 pandemic. We trained three types of machine learning models using the pre-COVID data and then compared model estimates with the recorded alcohol sales in March, April, May, and June 2020. The results based on the best RF models showed large deviations between model estimates and the recorded alcohol sales in some states and particularly in the month of March 2020. These large deviations suggest that the relation between alcohol sales and alcohol outlet visits might have changed in ways that are no longer captured by the trained model (e.g., due to people purchasing alcohol online or purchasing a large amount of alcohol in one visit). Such large deviations were observed in spirits and wine sales, but not in beer sales whose model estimates were still fairly close to the recorded data. In addition, geographic differences were again observed in this relation change, with stronger changes observed in some states (e.g., spirits sales in AK, FL, MO, and ND) but not in some other states (e.g., spirits sales in AR, IL, KY, and TX,). More research is necessary to further understand how exactly people might have changed their alcohol purchasing behavior.”

In page 19, authors highlighted that “While the primary purpose of the business shutdowns and stay-at-home orders was to reduce exposure to the virus, they can unintentionally result in greater alcohol consumption and may have the counterintuitive effect of increasing the exposure of some population groups to COVID-19 due to their risky behavior associated with more frequent or more acute intoxication. The changes we demonstrated regarding alcohol sales and visits to alcohol outlets suggest that public policies during times of pandemic may need to consider alcohol availability as a factor that influences public health above and beyond the reduction of exposure to COVID-19 at public drinking establishments.” How do the findings (particularly related to RQ3) point to the aforementioned conclusion?

Our response: We thank the reviewer for noticing this issue. In this version, we revised the text to enhance the connection between this conclusion and our experiment results (see Lines 649-661): “While the primary purpose of the business shutdowns and stay-at-home orders was to reduce exposure to the virus, they can also result in unintentional consequences, such as increased spirits and wine sales (as shown in Figs. 2 and 5) and increased visits to liquor stores (as shown in Fig. 9). Our geographical analysis also shows that the increases in spirits and wine sales were as large as 20-40% in multiple states (as shown in Figs. 4 and 6). These increased alcohol sales and liquor store visits can in turn lead to greater alcohol consumption and may even increase the exposure of some population groups to COVID-19 due to their increased visits to liquor stores. Additionally, if these increased liquor sales are indicative of increased drinking, individuals engaging in heavy drinking may be less likely to engage in safe behavioral strategies, such as social distancing and mask wearing, that are intended to protect them from the exposure to the virus (1,44). Our study suggests that public policies during a pandemic like COVID-19 may need to consider alcohol availability as a risk factor in addition to others that can affect public health.”

All in all, what are the important implications that authors would try to contribute after having incorporating those cutting-edge methodology, namely human mobility data and machine learning?

Our response: We thank the reviewer’s comment. In this version, we revised and enhanced our description of the contributions of this work in Lines 691-704: “Our contributions focused on three research questions regarding alcohol sales, alcohol outlet visits, and their relation respectively. First, our analysis showed some consistency with anecdotal reports in that the sales of spirits and wine indeed significantly increased since March 2020 compared to the same months in previous years; however, the increase was not homogeneous across different alcohol types, and in fact, the sales of beer largely decreased. Second, we found that people’s visits to liquor stores largely increased in the early months of the pandemic, but visits to other types of alcohol outlets (i.e., drinking places, breweries, and wineries) dramatically decreased. Third, we found that the relation between alcohol sales and alcohol outlet visits might have changed after the pandemic began in ways that can no longer be captured by machine learning models that were trained on alcohol outlet visits and other general information, due to possible reasons such as online alcohol purchase and stockpiling. In addition, our analysis showed geographic differences in alcohol sales, outlet visits, and their relation, which revealed the different responses to COVID-19 in terms of people’s alcohol purchasing behavior.”
